# Commentary: RNA editing with CRISPR-Cas13

**DOI:** 10.3389/fgene.2018.00134

**Published:** 2018-04-18

**Authors:** Ianis G. Matsoukas

**Affiliations:** Faculty of Health and Wellbeing, School of Sport and Biomedical Sciences, University of Bolton, Bolton, United Kingdom

**Keywords:** ADAR-editing, RNA editing, CRISPR, targeted gene repair, Deaminase, TadA

A paper recently published in Science (Cox et al., 2017) reports the possibility of editing RNA transcripts to alter their coding potential in a programmable manner. It is proposed that the RNA Editing for Programmable A to I Replacement (REPAIR), the new genome-editing technology that targets and alters RNA bases, offers a more temporary alternative to DNA editing.

## Programmable genome editing tools

Genome editing with programmable nucleases has become a powerful genetic tool. The term “programmable” refers to the ability to engineer the nuclease-based platforms for recognizing various target sites in different genomes. Many excellent reviews are available with regards to genome editing tools (Hsu et al., 2014; Kim and Kim, 2014; Cox et al., 2015; Kim, 2016). Therefore, the different genome editing-tools will not be discussed in great detail here.

**Figure 1:**
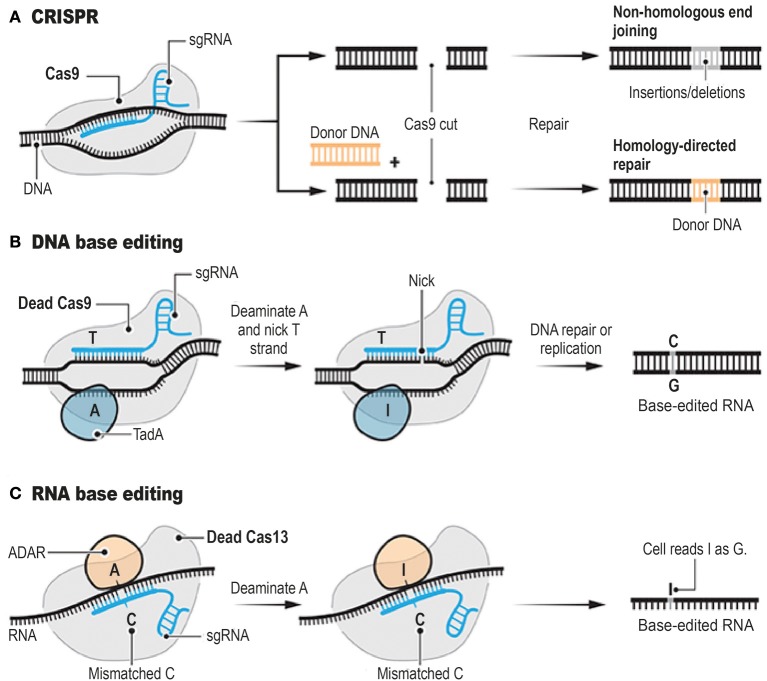
Programmable Genome Editing Tools. The conventional CRISPR DNA editor **(A)**. CRISPR relies on the ability of CRISPR sgRNAs to target the Cas9 endonuclease to precise genomic locations, where Cas9 introduces DSBs. Base editors borrow sgRNAs and Cas9 or other nucleases from CRISPR. However, base editors do not cut the double strand, but instead they chemically alter single bases with deaminase enzymes such as TadA (**B**, DNA base editor) and ADAR (**C**, RNA base editor). Adapted from Doudna and Charpentier (2014), Cohen (2017), Cox et al. (2017), and Gaudelli et al. (2017).

Genome editing tools include meganucleases (MN; Hafez and Hausner, 2012; Stoddard, 2014), zinc finger nucleases (ZFN; Carroll, 2011), transcription activator-like effector nucleases (TALENs; Boch et al., 2009; Boch, 2011), targetrons (Karberg et al., 2001), and the clustered regularly interspaced short palindromic repeat (CRISPR)-associated nuclease Cas9 (Bhaya et al., 2011; Jinek et al., 2012; Jiang et al., 2013). These genome editing tools can achieve precise genome modifications by inducing targeted DNA double-strand breaks (DSBs). CRISPR system has rapidly gone from being a niche technology to a mainstream method (Figure 1A). Interestingly, ongoing improvements of the CRISPR system have led to the development of powerful alternatives to standard CRISPR technology (Abudayyeh et al., 2017; Gaudelli et al., 2017, Figure 1B). CRISPR (Barrangou et al., 2007), relies on the ability of CRISPR single guide RNAs (sgRNAs) to target the Cas9 endonuclease to precise genomic locations, where Cas9 introduces DSBs (Hsu et al., 2013; Doudna and Charpentier, 2014). MNs, ZFNs, and TALENs achieve sequence-specific DNA-binding via protein-DNA interactions (Kim and Kim, 2014), whereas CRISPR and targetrons are RNA-guided systems (Zimmerly et al., 1995; Jiang et al., 2013). One crucial concern when applying these genome editing tools is the potential of cleavage at non-targeted sites. This episode can be lethal or generate undesirable permanent alterations of the nucleotide sequences.

## Exploiting a novel crispr system to target and edit RNA

RNA editing is a posttranscriptional process through which the cellular machineries can make discrete changes to specific nucleoside sequences within a RNA molecule (Gott and Emeson, 2000; Bass, 2002). In humans, the most common type of RNA editing is the conversion of adenosine to inosine (A → I; Hogg et al., 2011; Wulff et al., 2011; Gilbert et al., 2016; Zhao et al., 2017). This modification is mediated by two Adenosine Deaminases Acting on RNA (ADAR): ADAR1 and ADAR2. As I is read as guanosine by the splicing and translation apparatuses, ADARs can also amend splicing patterns and modify amino-acid sequences (Patterson and Samuel, 1995; Gott and Emeson, 2000; Desterro et al., 2003; Hogg et al., 2011; Nishikura, 2016).

Interestingly, a paper recently published in Science (Cox et al., 2017) reports the possibility of editing RNA transcripts to alter their coding potential in a programmable manner. Cox et al. (2017) named the new system “RNA Editing for Programmable A to I Replacement” (REPAIR; Figure 1C). This innovative article follows another important publication (Abudayyeh et al., 2017) from the same research group showing that a CRISPR system with an enzyme called Cas13a can target and cleave specific strands of RNA. Abudayyeh et al. (2017) identified the Cas13a from *Leptotrichia wadei* (LwaCas13a). LwaCas13a was heterologously expressed in mammalian and plant cells for targeted knockdown of either reporter or endogenous transcripts with comparable levels of knockdown as RNA interference, and improved specificity (Abudayyeh et al., 2017).

To create REPAIR, Cox et al. (2017) systematically profiled the CRISPR-Cas13 enzyme family for other potential “editor” candidates. They selected the Cas13b ortholog from *Prevotella* sp. P5-125 (PspCas13b), which was the most effective at inactivating RNA. They successfully engineered a deactivated variant of PspCas13b that still binds to specific nucleosides of RNA but lacks its “scissor-like” function. Then, the deactivated variant of PspCas13b was fused to ADAR2 deaminase domain, which is involved (with ADAR1) in the A → I conversion in RNA transcripts. Hence, in the novel system, the deactivated form of the Cas13b enzyme was able to recognize a target sequence of RNA, whereas the ADAR2 element was performing the base conversion without cleaving the transcript or relying on the native cellular apparatus.

Cox et al. (2017) modified REPAIR to improve its specificity by creating the REPAIRv1 system. To demonstrate the broad applicability of the REPAIRv1 system for RNA editing in mammalian cells, Cox et al. (2017) designed REPAIRv1 guides against two disease relevant mutations: 878G>A (AVPR2 W293X) in X-linked Nephrogenic diabetes insipidus, and 1517G>A (FANCC W506X) in Fanconi anemia. In the cell line with the DNA containing the anemia mutation, REPAIRv1 was able to correct 23% of the mutated RNA sequences. In the cell line containing the mutation causing the diabetes, REPAIRv1 was able to correct 35% of the mutated RNA sequences.

Interestingly, Cox et al. (2017) further modified REPAIRv1 to improve its specificity. The upgraded incarnation, REPAIRv2, consistently achieved the desired edit in 20–40%, and up to 51% of a targeted RNA without significant detection off-target activity. In addition, REPAIRv2 was able to reduce the detectable off-target edits from 18,385 to only 20 in the whole transcriptome, providing dramatically higher specificity than previously described RNA editing platforms (Stafforst and Schneider, 2012; Montiel-González et al., 2016).

## Concluding remarks

REPAIR presents a promising RNA editing platform with broad applicability for biotechnology research and therapeutics. Cox et al. (2017) demonstrated the use of the PspCas13b enzyme as both an RNA knockdown and RNA editing tool. Interestingly, the temporary nature of REPAIR-mediated edits will likely be useful for treating diseases caused by temporary alterations. For instance, Cas13b could be fused to a variety of editing enzymes that would allow a range of different sequence modifications.

The REPAIR system is an excellent research tool. Introducing specific sequence modifications into RNA molecules could allow to answer questions about alternative splicing mechanisms, translation, and even editing. In addition, by editing RNA rather than DNA, it might be possible to confer temporary, reversible genetic edits, rather than the CRISPR's permanent genome edits. This would allow the potential for temporal control over editing outcomes, as well as avoid the ethical issues that have arisen around genome editing.

However, due to RNA specific properties, the RNA base editors would have to be repeatedly administered to function as a therapeutic approach. In addition, despite the large number of recent findings and novelties to improve CRISPR via REPAIR (Cox et al., 2017), adenine base editors (Gaudelli et al., 2017), and other base editors (Komor et al., 2016), it could be several years before base-editing therapies enter clinical trials, and longer until it is clear whether the different base-editing strategies offer advantages over existing gene therapies.

## Author contributions

The author confirms being the sole contributor of this work and approved it for publication.

## Conflict of interest statement

The author declares that the research was conducted in the absence of any commercial or financial relationships that could be construed as a potential conflict of interest. The reviewer EP and handling Editor declared their shared affiliation.
